# Rescue of mitochondrial and neurite pathology in *SPG7* hereditary spastic paraplegia patient-derived cortical neurons

**DOI:** 10.1093/braincomms/fcag219

**Published:** 2026-06-25

**Authors:** David Neubauer, Carlotta Kaißer, Laura Krumm, Tom Boerstler, Klara Metzner, Thamer Al Hamad, Martin Böttcher, Simon Geißler, Iryna Prots, Philipp Arnold, Dimitrios Mougiakakos, Jürgen Winkler, Beate Winner, Martin Regensburger

**Affiliations:** Department of Stem Cell Biology, Friedrich-Alexander-Universität (FAU) Erlangen-Nürnberg, Erlangen 91054, Germany; Department of Stem Cell Biology, Friedrich-Alexander-Universität (FAU) Erlangen-Nürnberg, Erlangen 91054, Germany; Department of Molecular Neurology, FAU Erlangen-Nürnberg, Erlangen 91054, Germany; Department of Stem Cell Biology, Friedrich-Alexander-Universität (FAU) Erlangen-Nürnberg, Erlangen 91054, Germany; Department of Stem Cell Biology, Friedrich-Alexander-Universität (FAU) Erlangen-Nürnberg, Erlangen 91054, Germany; Department of Stem Cell Biology, Friedrich-Alexander-Universität (FAU) Erlangen-Nürnberg, Erlangen 91054, Germany; Department of Stem Cell Biology, Friedrich-Alexander-Universität (FAU) Erlangen-Nürnberg, Erlangen 91054, Germany; Health Campus for Inflammation, Immunity and Infection (GCI3), Otto-von-Guericke University Magdeburg, Magdeburg 39120, Germany; Department of Hematology, Oncology, Cell and Radiation Therapy, Otto-von-Guericke University Magdeburg, Magdeburg 39120, Germany; Department of Stem Cell Biology, Friedrich-Alexander-Universität (FAU) Erlangen-Nürnberg, Erlangen 91054, Germany; Department of Stem Cell Biology, Friedrich-Alexander-Universität (FAU) Erlangen-Nürnberg, Erlangen 91054, Germany; Department of Conservative Dentistry and Periodontology, FAU Erlangen-Nürnberg, Erlangen 91054, Germany; Institute of Functional and Clinical Anatomy, FAU Erlangen-Nürnberg, Erlangen 91054, Germany; Health Campus for Inflammation, Immunity and Infection (GCI3), Otto-von-Guericke University Magdeburg, Magdeburg 39120, Germany; Department of Hematology, Oncology, Cell and Radiation Therapy, Otto-von-Guericke University Magdeburg, Magdeburg 39120, Germany; Department of Molecular Neurology, FAU Erlangen-Nürnberg, Erlangen 91054, Germany; Department of Stem Cell Biology, Friedrich-Alexander-Universität (FAU) Erlangen-Nürnberg, Erlangen 91054, Germany; Center for Rare Diseases Erlangen (ZSEER), University Hospital Erlangen, Erlangen 91054, Germany; Department of Stem Cell Biology, Friedrich-Alexander-Universität (FAU) Erlangen-Nürnberg, Erlangen 91054, Germany; Department of Molecular Neurology, FAU Erlangen-Nürnberg, Erlangen 91054, Germany; Center for Rare Diseases Erlangen (ZSEER), University Hospital Erlangen, Erlangen 91054, Germany; Deutsches Zentrum Immuntherapie (DZI), University Hospital Erlangen, Erlangen 91054, Germany

**Keywords:** SPG7, hereditary spastic paraplegia (HSP), cortical motor neuron, induced pluripotent stem cells, mitochondria

## Abstract

Biallelic pathogenic variants in *SPG7* are a frequent cause of hereditary spastic paraplegia leading to progressive disability due to a length-dependent degeneration of cerebellar and cortical projection neurons. While underlying mechanisms have been linked to impaired mitochondrial function, no disease-modifying therapy is available. We generated induced pluripotent stem cell–derived cortical neurons from SPG7 patients with non-sense or truncating variants and from matched controls. We performed detailed phenotyping of neuronal differentiation, as well as mitochondrial and neuritic morphology and function. We explored the effects of Bz-423, a modulator of the mitochondrial permeability transition pore, as a potential rescue of SPG7-specific cellular phenotypes. We successfully differentiated SPG7 patient-derived neurons, without quantitative differences in differentiation compared with controls. However, we delineate neurite-specific aberrations of mitochondrial morphology and ultrastructure. Moreover, anterograde axonal mitochondrial transport was impaired in SPG7. Exposure to Bz-423 rescued ultrastructural and functional phenotypes. In summary, our data show impaired neuritic mitochondria in a patient-specific human model, and we here demonstrate for the first time beneficial effects of Bz-423 on neuritic ultrastructure and function in a human neuronal SPG7 system. Moreover, we identify the mitochondrial permeability transition pore as a molecular target to rescue phenotypes also in carriers of non-sense or truncating *SPG7* variants.

## Teaser

Neubauer *et al*. report on the phenotypes of cortical motor neurons derived from *SPG7* hereditary spastic paraplegia patient induced pluripotent stem cells, including abnormal axonal mitochondrial morphology and decreased mitochondrial transport. Modulation of the mitochondrial permeability transition pore led to a partial rescue of the observed phenotypes.

## Graphical Abstract

**Graphical Abstract:**
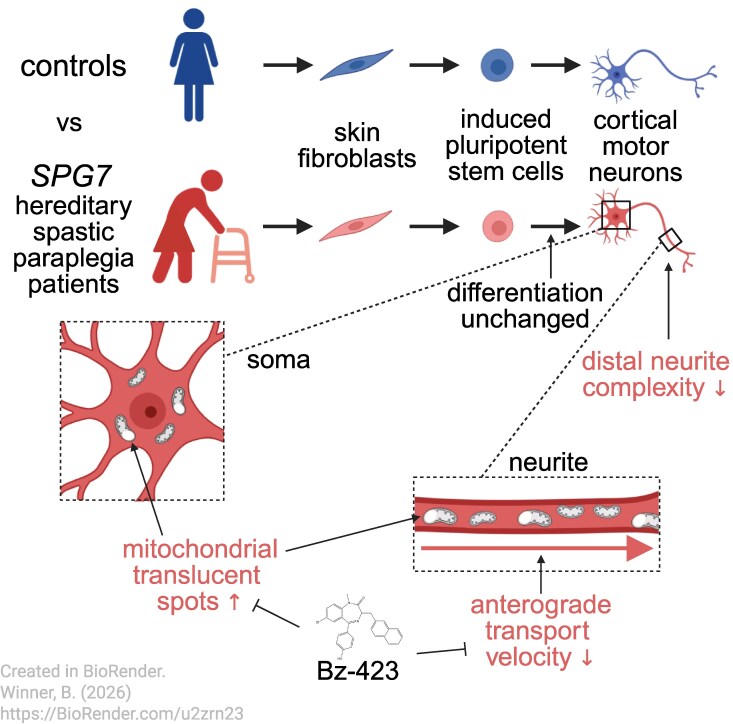

## Introduction

Hereditary spastic paraplegias (HSPs) are a heterogeneous group of monogenic neurodegenerative disorders characterized by an axonal dying-back of cortical layer V motor neurons. The core symptoms are lower limb spasticity and weakness. To date, over 80 genes have been identified as causative for HSP. Pathogenic variants in *SPG7* cause a distinct subtype of autosomal-recessive HSP (SPG7-HSP). In addition to progressive spasticity of the lower extremities, SPG7-HSP encompasses a spectrum of cerebellar ataxia, optic atrophy and peripheral neuropathy, and no causal therapy is available to date.^1,2^ The precise cellular mechanism of the axonal dying-back is yet unknown but has been linked to mitochondrial dysfunction. *SPG7* encodes paraplegin, a mitochondrial protein containing an AAA (ATPase associated with diverse cellular activities) protease domain. The AAA protease localizes to the inner mitochondrial membrane and acts in protein quality control, as well as regulation of respiratory chain activity.^3-5^ Previous fly and mouse models of SPG7-HSP exhibited deficits in axonal transport as a potential correlate of axonal degeneration and mitochondrial dysfunction,^6,7^ but more detailed analyses of patient-derived neurons and the neurite-mitochondria interaction are lacking. Patient-specific induced pluripotent stem cells (iPSCs) and neurons recapitulate the genetic background of each individual genotype *in vitro* and are increasingly being used to elucidate disease mechanisms and to establish new therapeutic strategies.^8^

Using iPSC, we here established a neuronal SPG7 patient-derived *in vitro* model, in order to (i) characterize neuronal differentiation and mutant paraplegin expression, (ii) analyse mitochondrial morphology, (iii) quantify neurite growth and axonal transport and (iv) define patient-specific *in vitro* outcome parameters for potential therapies. We provide evidence that paraplegin-mutant or paraplegin-deficient iPSC-derived cortical neurons exhibit abnormal mitochondrial ultrastructure, as well as impaired axonal transport. Moreover, we report the beneficial effects of the mitochondrial membrane pore modulator Bz-423 on SPG7-HSP-related mitochondrial phenotypes.

## Materials and methods

### Generation of induced pluripotent stem cells

All participants provided written informed consent, and all experiments received approval from the local institutional review board (approval no. 259_17B: ‘Biobank zur Untersuchung von Biomarkern und Generierung humaner Zellmodelle bei Neurologischen Erkrankungen’, ethics committee of the Friedrich-Alexander-Universität Erlangen-Nürnberg, Erlangen, Germany). This study included three patients with biallelic pathogenic variants in the *SPG7* gene and three age- and sex-matched controls (Supplementary Table 1). In each patient, at least one variant was a non-sense or truncating variant.

### Cortical neuron differentiation

Control and patient iPSCs were differentiated into neural progenitor cells (NPCs) and cortical neurons following an adapted dual SMAD inhibition–based small molecule monolayer protocol as described earlier.^9,10^ For details on the differentiation protocol and western blot, see Supplementary Methods.

### Pharmacological modulation

Bz-423 was dissolved at 300 μM in dimethyl sulphoxide (DMSO) and added to the cell culture media with a final concentration of 300 nM (#5791, Tocris). Cells were treated with Bz-423 throughout differentiation.

### Statistical analysis

Statistical analysis was performed using R (version 2025.05.0+496). To analyse the effect of genotype (control versus SPG7) for the different readouts, a linear mixed-effects model was employed to account for the interrelation of two clones per donor. To this end, we used the lmer function (lmerTest package). The model included genotype (control versus SPG7) as a fixed effect and nested random intercepts for clone within donor to account for the hierarchical structure of the data [model formula: parameter ∼ genotype + (1|donor/clone)]. Model fitting was performed using restricted maximum likelihood. Degrees of freedom and *P*-values for the fixed effect of genotype were estimated using Satterthwaite’s approximation. For the assessment of treatment effects, i.e. when comparing four groups, treatment (DMSO versus Bz-423) was used as a second fixed effect. Estimated marginal means were computed using the emmeans package with Kenward–Roger degrees-of-freedom approximation and Tukey adjustment for multiple comparisons.

## Results

### SPG7 patient induced pluripotent stem cells differentiate into cortical neurons

**Figure 1:**
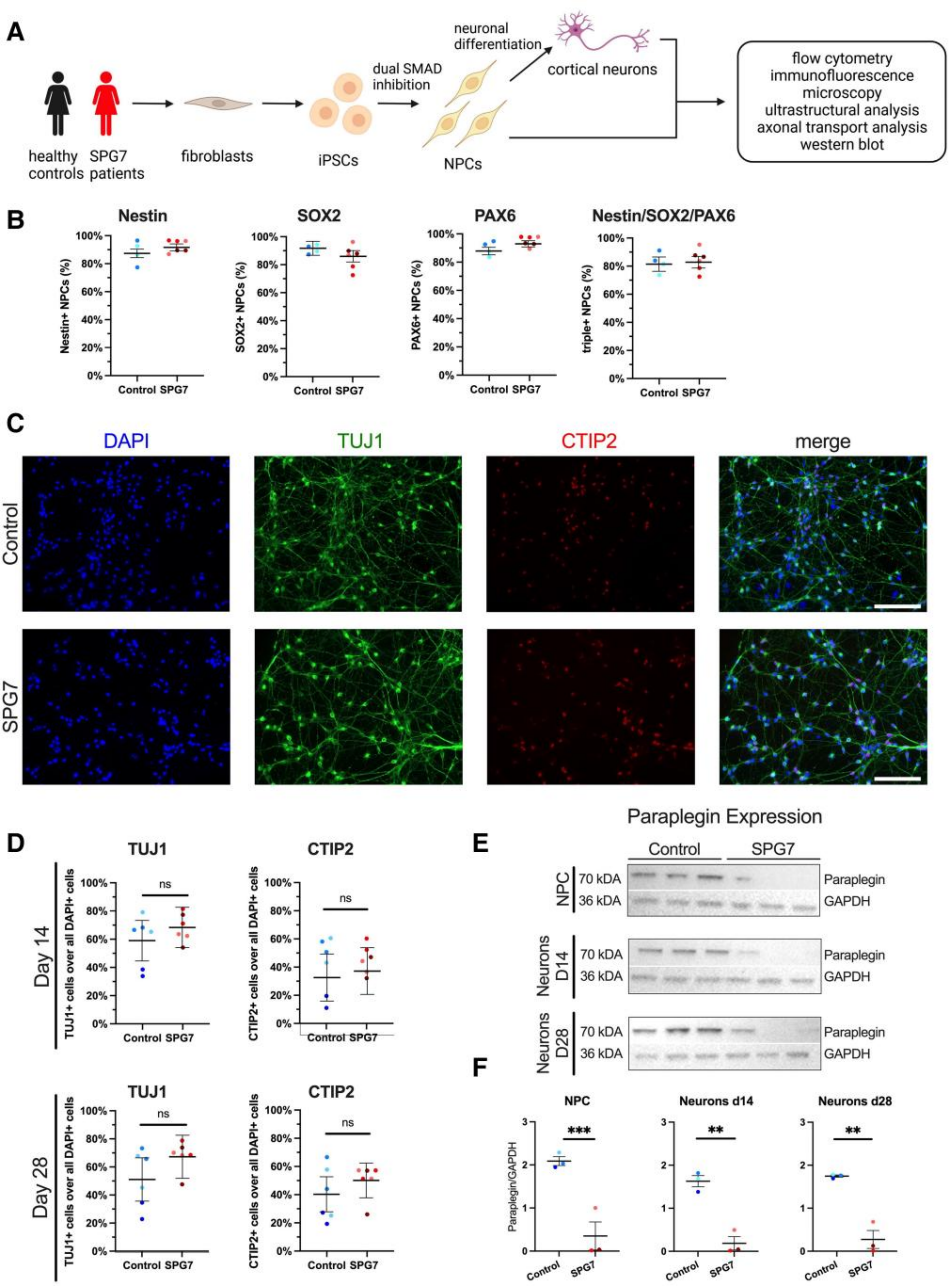
**Differentiation and characterization of control and SPG7 patient iPSC-derived cortical neurons.** (**A**) Paradigm of differentiation and overview of phenotypic characterizations, created with BioRender.com. (**B**) Characterization of NPCs by flow cytometry for the markers Nestin, SOX2, PAX6 and Nestin/SOX2/PAX6 triple-positive cells, showing no significant differences between controls and SPG7, according to a linear mixed-effects model. *n*_Control_ = 4 iPSC clones derived from 2 donors, *n*_SPG7_ = 6 iPSC clones derived from 3 donors. Per clone, flow cytometry was performed on 10 000 cells. Each dot represents results from one iPSC clone. Each donor is coded by a separate colour. (**C**) Representative immunofluorescence images in control and SPG7 cortical neurons (CNs) at 14 days of differentiation from NPCs (scale bar, 100 μm). (**D**) Quantification of CN differentiation for TUJ1 and CTIP2 at 14 and 28 days of neuronal differentiation, comparing controls with SPG7 by linear mixed-effects models. *n*_Control_ = 6 iPSC clones derived from 3 donors, *n*_SPG7_ = 6 iPSC clones derived from 3 donors. Per clone, at least six fields of view were quantified, obtained from at least two experimental replicates. Each dot represents results from one iPSC clone. Each donor is coded by a separate colour. (**E**) Analysis of paraplegin expression by western blot at the NPC stage, after 14 days and after 28 days of neuronal differentiation. (**F**) Quantification of relative paraplegin expression normalized for GAPDH and Control NPC. Groups were compared by a linear mixed-effects model. *n*_Control_ = 3 iPSC clones derived from 3 donors, *n*_SPG7_ = 3 iPSC clones derived from 3 donors. Each dot represents the relative band intensity from one iPSC clone. ns, not significant; **P < 0.01; ***P < 0.001. Clone-specific data of **B**, **D** and **F** are shown in Supplementary Fig. 1.

We recruited three patients diagnosed with a genetically confirmed SPG7-HSP and at least one non-sense or truncating variant. We matched them with three controls based on age and sex (Supplementary Table 1). All patients displayed typical features of spastic paraplegia as well as mild cerebellar signs. To generate cortical neurons, fibroblasts underwent reprogramming into iPSCs. Subsequently, two iPSC clones per individual were differentiated (paradigm in Fig. 1A). The pluripotency of iPSCs was confirmed by analysis of Tra-1-60 expression, with no discernible differences between SPG7 and control iPSCs (Supplementary Fig. 1A). All lines were negative for pathogenic large copy number variations (Supplementary Fig. 5). NPCs were generated using a dual SMAD inhibition–based monolayer differentiation protocol. Notably, there were no significant differences in differentiation capacity, as evidenced by high rates of PAX6+/NESTIN+/SOX2+ cells (Fig. 1B; Supplementary Fig. 1B). Subsequently, we directed the differentiation of these NPCs into forebrain patterned neurons using Brain-Derived Neurotrophic Factor, Glial cell line-Derived Neurotrophic Factor and cyclic Adenosine Monophosphate. The efficacy of neuronal differentiation was assessed at both 14 and 28 days by quantifying the expression of βIII-tubulin-positive neurons [positive for tubulin antibody clone J1 (TUJ1)] and chicken ovalbumin upstream promoter transcription factor interacting protein 2 (CTIP2)–positive motor neurons (Fig. 1C and D; Supplementary Fig. 1C). No significant differences in differentiation were observed. Neuronal loss within the SPG7 cultures was not evident (*P* > 0.05 for quantification of TUJ1 and CTIP2, respectively). At Day 14 of differentiation, ∼60% of cells in both control and SPG7 iPSC-derived cortical neurons were positive for TUJ1, a percentage which persisted after 28 days (Fig. 1D). About 50% of cells expressed the cortical neuron marker CTIP2, suggesting that cortical neuron generation reached a plateau by Day 14. We subsequently aimed to characterize the expression of paraplegin during differentiation using Western blotting. Matching the fact that at least one of the *SPG7* gene variants was a non-sense or frameshift variant for each patient of our cohort (Supplementary Table 1), we observed a significant reduction of paraplegin expression both at the NPC and at the differentiated neuron stage after 14 and 28 days of differentiation (Fig. 1E and F). We observed no additional bands that might indicate truncated protein (Supplementary Fig. 1D).

### Neuritic pathology in SPG7 neurons

**Figure 2:**
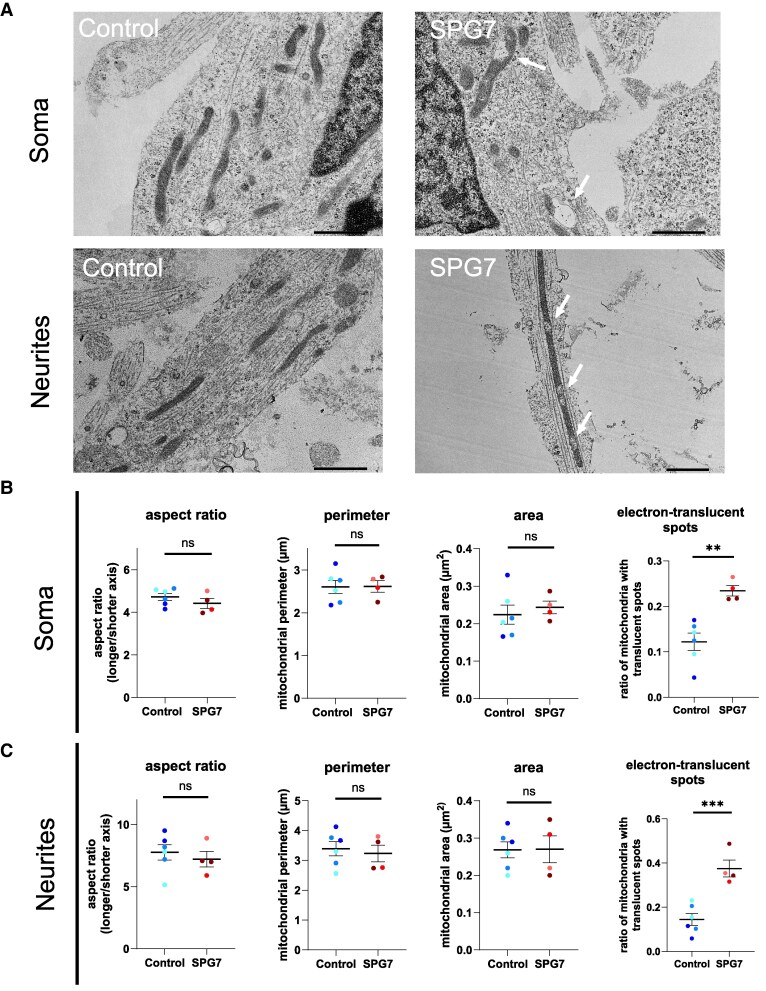
**Analysis of mitochondrial morphology.** (**A**) Representative ultrastructural images of mitochondria located in the soma (upper row) and in neurites (lower row) were acquired by electron microscopy of control and SPG7 cortical neurons. Scale bars, 1 μm. Arrows indicate electron translucent spots. (**B**) Quantification of morphological parameters of mitochondria located in the soma including aspect ratio (defined as the ratio of the longest to the shortest mitochondrial axis), perimeter, circularity and the ratio of mitochondria containing electron translucent spots. (**C**) Quantification of morphology parameters of mitochondria localized within neurites including aspect ratio, perimeter and the ratio of mitochondria with electron translucent spots. For **B** and **C**, each dot indicates the mean of one iPSC clone. Groups were compared by linear mixed-effects models. *n*_Control_ = 6 iPSC clones derived from 3 donors, *n*_SPG7_ = 4 iPSC clones derived from 3 donors. Per clone, at least 30 mitochondria were quantified, obtained from at least 10 somata obtained in 2 technical replicates. Each dot represents results from one iPSC clone. Each donor is coded by a separate colour. ns, not significant; *P < 0.05; **P < 0.01; ***P < 0.001. Clone-specific data of **B** and **C** are shown in Supplementary Fig. 2.

Next, we performed an ultrastructural analysis of mitochondria in cortical neurons. We first analysed mitochondria within the soma (Fig. 2A). There were no differences regarding mitochondrial aspect ratio, mitochondrial perimeter and mitochondrial area, respectively (Fig. 2B; Supplementary Fig. 2A). We observed frequent structural abnormalities of mitochondria, i.e. electron translucent spots of the mitochondrial matrix, compatible with local perturbation of the cristae structure. These electron translucent spots were present in the soma of a mean of 23.5% (SPG7) versus 12.2% (control) mitochondria (Fig. 2B; Supplementary Fig. 2A). We next focused on mitochondria localized within neurites (Fig. 2A and C). There were no significant differences regarding mitochondrial dimension parameters (Fig. 2C; Supplementary Fig. 2B). Electron translucent spots within mitochondria were also present within neurites, and these regions were significantly more frequent in neurites derived from SPG7 patients compared with those from controls (37.5% SPG7 versus 14.5% control; Fig. 2C; Supplementary Fig. 2B).

### Impaired axonal transport in SPG7 patient-derived neurons

**Figure 3:**
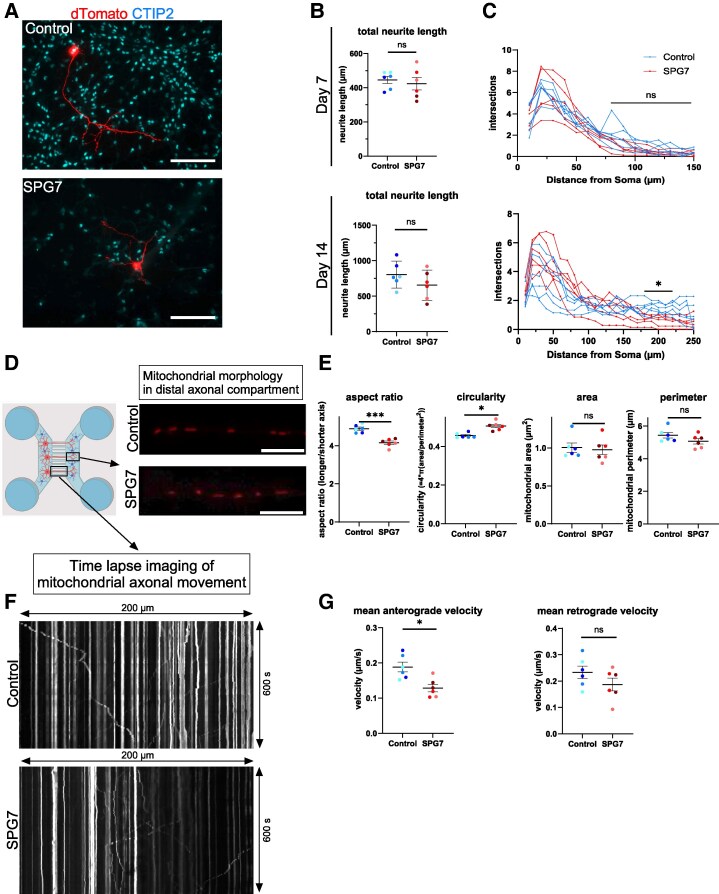
**Analysis of neurite outgrowth and mitochondrial transport.** (**A**) Representative images of control (upper image) and SPG7 (lower image) cortical neurons after 14 days of neuronal differentiation stained for CTIP2 expression after transfection with a dTomato expressing plasmid to label single neurons. Scale bars, 100 μm. (**B**) Quantification of total neurite length after 7 days (upper graph) and after 14 days (lower graph) of neuronal differentiation. Groups were compared by linear mixed-effects models. *n*_Control_ = 6 iPSC clones derived from 3 donors, *n*_SPG7_ = 6 iPSC clones derived from 3 donors. Per clone, complete neurite morphology from at least eight neurons was quantified. Each dot represents the mean value of one iPSC clone, and each donor is coded by a separate colour. (**C**) Sholl analysis plots of cortical neurons after 7 days (upper graph) and 14 days (lower graph) of neuronal differentiation. Groups were compared by linear mixed-effects models at each Sholl radius, analysed on the same dataset as in **B**. Each line represents the mean of a single iPSC clone, with at least eight neurons quantified per clone. (**D**) Paradigm of the microfluidic chamber set-up for the analysis of mitochondrial transport and mitochondrial morphology in the distal axonal compartment. Representative images of control and SPG7 mitochondria located in the distal axonal compartment, labelled by lentiviral expression of mitoDsRed. Scale bars, 10 μm. (**E**) Quantification of the mitochondrial morphological parameters aspect ratio, circularity, area and perimeter. Groups were compared by linear mixed-effects models. *n*_Control_ = 6 iPSC clones derived from 3 donors, n_SPG7_ = 6 iPSC clones derived from 3 donors. Per clone, at least 30 axon compartment mitochondria from 2 microfluidic chambers were quantified, obtained at 2 experimental timepoints. Each dot represents the mean of one iPSC clone, and each donor is coded by a separate colour. (**F**) Representative kymographs of control and SPG7 axonal mitochondria over an axon length of 200 μm (horizontal axis) and a duration of 600 s (vertical axis). (**G**) Quantification of anterograde and retrograde mitochondrial transport velocity. Analyses were performed after 14 days of neuronal differentiation. Groups were compared by linear mixed-effects models. *n*_Control_ = 6 iPSC clones derived from 3 donors, *n*_SPG7_ = 6 iPSC clones derived from 3 donors. Per clone, at least 10 mitochondria from 2 microfluidic chambers were quantified, obtained at 2 experimental timepoints. Each dot represents the mean of one iPSC clone, and each donor is coded by a separate colour. ns, not significant; *P < 0.05; ***P < 0.001. Clone-specific data of **B**, **E** and **G** are shown in Supplementary Fig. 3.

In light of the observed mitochondrial ultrastructural aberration, we next sought to characterize morphology and function of the neuritic compartment. Neurite outgrowth of cortical neurons was assessed by transfection-based fluorescent labelling of single cells (Fig. 3A). We analysed neurite outgrowth at two time points of differentiation, i.e. on Day 7 and on Day 14. After 7 days of cortical neuronal differentiation, there were no significant differences between control and SPG7 cortical neurons regarding total neurite length and Sholl analysis (Fig. 3B and C; Supplementary Fig. 3A). After 14 days of differentiation, there was no statistical difference in total neurite length albeit a trend of decreased neurite length in SPG7 (Fig. 3B; Supplementary Fig. 3A). Sholl analysis revealed about three to four primary neurites per neuron, and there was a significant reduction of the number of intersections at a radius >150 μm in SPG7 (Fig. 3C). This indicates a specific reduction of the longest neurites in SPG7 cortical neurons, but rather preserved total neurite length per neuron. In order to focus on the axonal compartment, we used a microfluidic chamber set-up where cells were seeded at the NPC stage and differentiated into cortical neurons. Mitochondria were labelled with a lentivirus overexpressing mitoDsRed (Fig. 3D). We focused on mitochondrial morphology specifically in the very distal axonal compartment. There was a significant reduction in the aspect ratio of SPG7 distal mitochondria (Fig. 3E). Accordingly, mitochondrial circularity was increased in SPG7 (Fig. 3E). Altogether, this indicates fragmented and more circular distal mitochondria in SPG7. Mitochondrial perimeter and area did not differ between control and SPG7 distal mitochondria (Fig. 3E). This observation corresponds to the aforementioned loss of long-growing neurites in SPG7 cortical neurons (Fig. 3C). To assess whether these morphological changes were accompanied by alterations in mitochondrial transport, we analysed mitochondrial transport in the microfluidic chamber set-up after 14 and 28 days of neuronal differentiation (Fig. 3F). Mitochondrial movements were classified into anterograde and retrograde. There was a profound reduction of mean anterograde mitochondrial transport velocity in SPG7 cortical neurons on Day 14 of differentiation (37% reduction compared with controls, Fig. 3G; Supplementary Fig. 3C). This reduction partly recovered until Day 28 (Supplementary Fig. 3D). Retrograde transport velocity, in turn, was unchanged when comparing control and SPG7 neurons (Fig. 3G; Supplementary Fig. 3D). Likewise, the ratio of anterograde over all movements was unchanged between SPG7 and controls at both experimental time points (Supplementary Fig. 3C and D).

### Bz-423 rescues axonal dysfunction in SPG7 patient neurons

**Figure 4:**
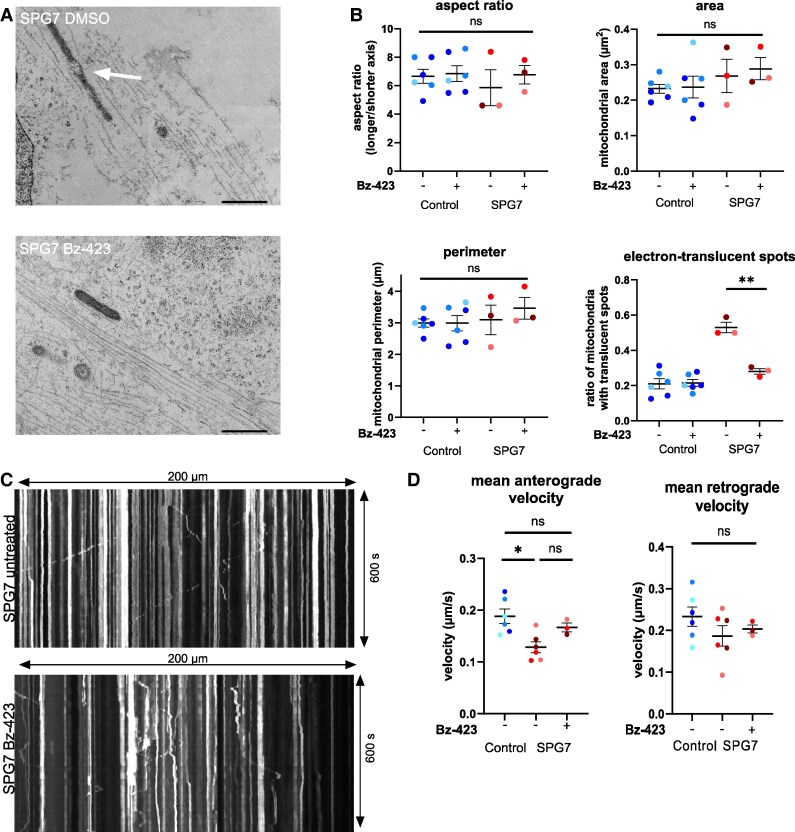
**Pharmacological rescue of SPG7 neuronal phenotypes by Bz-423.** (**A**) Representative ultrastructural images of mitochondria of DMSO-treated (upper image) and Bz-423-treated (lower image) SPG7 cortical neurons (CNs). Scale bars, 1 μm. The arrow indicates an electron translucent spot. (**B**) Quantification of the morphological parameters aspect ratio, area, perimeter and the ratio of mitochondria with electron translucent spots. SPG7-DMSO was compared with SPG7 Bz-423 by linear mixed-effects models with Tukey adjustment for between-group comparisons. *n*_Control_ = 6 iPSC clones derived from 3 donors (per treatment condition), *n*_SPG7_ = 3 iPSC clones derived from 3 donors (per treatment condition). Per clone, at least 10 mitochondria were quantified, obtained from 2 technical replicates. Each dot represents results from one iPSC clone. Each donor is coded by a separate colour. (**C**) Representative kymographs of untreated (upper image) and Bz-423-treated (lower image) neurites of SPG7 CNs over an axon length of 200 μm (horizontal axis) and a duration of 600 s (vertical axis). (**D**) Quantification of anterograde (left graph) and retrograde mitochondrial transport velocity (right graph). Analysis was performed by a linear mixed-effects model with Tukey adjustment for between-group comparisons. *n*_Control-untreated_ = 6 iPSC clones derived from 3 donors, *n*_SPG7-untreated_ = 6 iPSC clones derived from 3 donors, *n*_SPG7-treated_ = 3 iPSC clones derived from 3 donors. Per clone, at least 10 total mitochondria from 2 microfluidic chambers at 2 experimental timepoints were quantified. Each dot represents the mean of one iPSC clone, and each donor is coded by a separate colour. *P < 0.05. Clone-specific data of **B** and **D** are shown in Supplementary Fig. 4.

Bz-423 is a benzodiazepine-derived small molecule, which modulates the opening of the mitochondrial permeability transition pore (mPTP).^11^ In a previous study, Bz-423 improved the motor phenotype of Spg7^−/−^ mice and normalized firing patterns of derived primary cortical neurons.^12,13^ In light of the neuritic phenotypic alterations of our neuronal model, we next tested the effects of Bz-423 on SPG7 versus control cortical neurons. We used Bz-423 at a concentration of 300 nM throughout differentiation which did not show apoptotic effects (Supplementary Fig. 4A). Ultrastructural analysis of neuritic mitochondria indicated an increase in electron translucent mitochondrial spots in SPG7 versus controls under DMSO control conditions, whereas neuritic mitochondrial morphology was unchanged (Fig. 4A and B; Supplementary Fig. 4B). Bz-423-treated SPG7 cortical neurons, however, showed a significantly reduced ratio of mitochondria containing electron translucent spots, when compared with DMSO-treated SPG7 neurons.

In addition, there was a significant effect of Bz-423 on total neurite length and distal neurite complexity of SPG7 neurons (Supplementary Fig. 4C and D). However, this finding has to be interpreted with caution since baseline total neurite length showed no significant reduction in untreated SPG7 neurons (Fig. 3B).

Finally, we assessed the effect of Bz-423 on impaired axonal mitochondrial transport in SPG7 (Fig. 4C). Bz-423 treatment led to a rescue of impaired anterograde transport velocity of mitochondria in SPG7 (Fig. 4D). A subanalysis of these anterogradely moving mitochondria according to their velocity revealed that predominantly fast-moving mitochondria (velocity >0.3 μm/s) were reduced in SPG7, whereas the frequency of slow movements (velocity <0.1 μm/s) was increased accordingly (Supplementary Fig. 4F and G). Application of Bz-423, in turn, normalized velocity distribution to control levels.

## Discussion

In this study, we examined the mitochondrial and neuronal phenotype associated with *SPG7*-related HSP using iPSC-derived cortical neurons from SPG7 patients. We demonstrate mitochondrial translucent spots as well as significantly impaired anterograde axonal mitochondrial transport, rescued by application of the mPTP modulator Bz-423.

A previous study performed in patient-derived olfactory mucosa cells described increased expression levels of paraplegin, in line with the exclusive recruitment of missense variant carriers, including p.Ala510Val.^14^ Compared with our study showing a loss of paraplegin protein, the recruitment of biallelic missense paraplegin variant carriers and the subsequent compensatory increased paraplegin expression levels may partly explain different phenotypes. In this context, our report adds complementary data showing that patient iPSC-derived motor neurons represent a valid model not only for missense *SPG7* variants, but also for truncating *SPG7* variants associated with loss of paraplegin protein. The *in vitro* phenotypes of neurite outgrowth reported for the missense variant carrier cohort tended to be stronger than those observed for our cohort.^13^ In addition to different genotypes, lower throughput methods (single-cell labelling, ultrastructural analysis) used in our study and later timepoints of analysis might have led to decreased statistical power. Paraplegin itself is involved in the degradation of respiratory chain Complex I leading to an increased oxygen consumption in SPG7-HSP models, including patient fibroblasts and olfactory neurosphere derived cells.^6,14,15^ Future studies should aim to comprehensively characterize mitochondrial function in patient-derived neuronal cells at different developmental stages and in direct conversion models, which may circumvent potential metabolic reset effects of iPSC reprogramming.

We provide evidence that SPG7 cortical neurons exhibit abnormal mitochondrial morphology, consistent with previous reports in Drosophila, mouse and human olfactory mucosal cells.^6,7,14^ These aberrations were characterized by mitochondrial swellings and electron translucent spots indicating disorganized cristae structure. HSPs including SPG7-related HSP exhibit a neuronal ‘dying-back’ gradient, meaning neurodegeneration starts at the distal end of neurites and progresses towards the soma. Our findings of aberrant mitochondrial morphology with a length-dependent gradient may thus be an early event inducing neuronal dying-back, which was previously observed in two Spg7^−/−^ mouse models.^7,16^ Mitochondrial DNA depletion was described in muscle biopsies of SPG7 patients.^17,18^ In contrast, we did not detect differences in mitochondrial DNA content in SPG7 primary fibroblasts (data not shown).

Defects in organelle trafficking and axonal transport of mitochondria are frequently involved in the pathogenesis of neurodegenerative diseases. Impaired axonal transport was also detected in iPSC-derived neurons of other subtypes of HSP, including SPG4, SPG11 and SPG15.^19-21^ We confirm compromised axonal transport of mitochondria in SPG7 cortical neurons, which specifically affected anterograde events. This finding matches previous reports on SPG7 animal models, which also exhibited a specific deficiency of anterograde axonal transport.^6,7^ Yet, the precise mechanisms leading to this mitochondrial trafficking phenotype remain unknown. Organelle transport, for example, might be mechanically affected by dysmorphic mitochondria and accumulation of neurofilaments, leading to axonal swellings.^7^ Alternatively, a functional mitochondrial impairment may lead to the downstream transport phenotype.

Previously, SPG7 mitochondria were linked to increased intra-mitochondrial Ca^2+^ levels.^22,23^ To regulate mitochondrial motility in neurons, the protein Miro1 assembles with Trafficking kinesin-binding proteins 1 and 2 and the motor proteins Kinesin/Dynein. This motor/adaptor complex is tightly regulated by cytoplasmic and mitochondrial matrix Ca^2+^ levels. A previous study characterized an inverse correlation between mitochondrial matrix Ca^2+^ levels and average mitochondrial transport velocity in mouse hippocampal neurons, suggesting an intra-mitochondrial Ca^2+^ threshold blocking mitochondrial movements.^24^ Hence, mitochondrial transport in SPG7 cortical neurons may be influenced by both mechanical and mitochondrial Ca^2+^ disequilibrium reasons.

Additionally, our data show indications of an altered distal neurite outgrowth in SPG7 cortical neurons. While total neurite length was not significantly changed, Sholl analysis showed a distal neurite complexity phenotype, and we observed significantly impaired anterograde axonal transport. Neurite growth is an ATP-dependent process. Due to the deficiency in anterograde axonal transport, a lack of ATP in the distal neuritic compartment might be the limiting factor of local neurite growth, compatible with the finding of fragmented mitochondrial morphology in distal neurites.

Several proteins are involved in both mitochondrial Ca^2+^ uptake and release. Paraplegin itself is involved in mPTP function, either as a direct component or as a modulator.^12,22,23^ Physiologically, the mPTP opens upon increasing intra-mitochondrial Ca^2+^ levels, resulting in Ca^2+^ release and therefore preventing mitochondria from Ca^2+^-related toxicity.^25^ One key regulator of mPTP is Cyclophilin D (CypD), a mitochondrial peptidyl-prolyl cis-trans isomerase, modulating mPTP opening by sensitization to mitochondrial Ca^2+^ levels.^26-28^ Inversely, lack of CypD desensitizes mPTP opening to mitochondrial Ca^2+^ levels.^26,29^ In the CypD-null mouse model, mitochondria require double the levels of mitochondrial Ca^2+^ to initiate mPTP opening.^30^ Bz-423 mimics the function of CypD, thus decreasing the mPTP opening threshold and potentially preventing mitochondria from Ca^2+^-related toxicity.^11,31,32^

In this study, we provide evidence that treatment with Bz-423 leads to a profound improvement of mitochondrial morphology and transport velocity in SPG7 patient-derived cortical neurons. These data add to the previously reported beneficial effects of Bz-423 in *SPG7* missense mutation carrier iPSC-derived cortical neurons.^13^ While this previous report focused on neurite and mitochondrial morphology analysed by fluorescent labelling, which were rescued after 7 days of Bz-423 exposure, our report adds functional data on axonal transport and ultrastructural evidence of intra-mitochondrial abnormalities. Moreover, our data suggest that mPTP-directed therapeutic strategies would also be applicable for carriers of truncating *SPG7* variants. Ultrastructural analysis identified an increased number of aberrant mitochondria exhibiting electron translucent spots in SPG7. This supports a functional connection of these phenotypes with dysregulated mitochondrial matrix Ca^2+^ signalling. Although we did not directly determine neuronal mitochondrial Ca^2+^, Bz-423 may preserve physiological intra-mitochondrial Ca^2+^, reduce Ca^2+^-mediated stress and therefore increase mitochondrial longevity. In light of the length- and differentiation-dependent neuritic phenotypes in SPG7 cortical neurons, Bz-423 might lead to an improvement of mitochondrial function in distal neurites. Additionally, Bz-423 rescued impaired axonal organelle trafficking. Future experiments should specifically address these aspects in compartmentalized culture systems such as microfluidic chambers combined with functional Ca^2+^ assays. Moreover, application of Bz-423 improved neurite outgrowth and rescued the number of long neurites to healthy control levels, which could be a consequence of both accelerated anterograde axonal transport of mitochondria and improved function of distal neuritic mitochondria.

While our results of beneficial effects of Bz-423 confirm its action in SPG7 patient-derived neurons and validate the mPTP as a therapeutic target, the translational potential of Bz-423 itself is limited due to its effects on superoxide production and its pro-apoptotic effects at higher doses,^33,34^ asking for future drug development targeting mPTP modulation in SPG7.

## Supplementary Material

fcag219_Supplementary_Data

## Data Availability

Data will be available beginning 3 months and ending 5 years following article publication. Data will be shared with researchers who provide a methodologically sound proposal. Data will be shared for analyses to achieve the aims in the approved proposal. Proposals should be directed to the corresponding author; to gain access, data requestors will need to sign a data access agreement.

## References

[1] Schüle R, Wiethoff S, Martus P, et al Hereditary spastic paraplegia: Clinicogenetic lessons from 608 patients. Ann Neurol. 2016;79(4):646–658. 26856398 10.1002/ana.24611

[2] Erfanian Omidvar M, Torkamandi S, Rezaei S, et al Genotype–phenotype associations in hereditary spastic paraplegia: A systematic review and meta-analysis on 13,570 patients. J Neurol. 2019;268(6):2065–2082. 31745725 10.1007/s00415-019-09633-1

[3] Casari G, De Fusco MD, Ciarmatori S, et al Spastic paraplegia and OXPHOS impairment caused by mutations in paraplegin, a nuclear-encoded mitochondrial metalloprotease. Cell. 1998;93(6):973–983. 9635427 10.1016/s0092-8674(00)81203-9

[4] Nolden M, Ehses S, Koppen M, Bernacchia A, Rugarli EI, Langer T. The m-AAA protease defective in hereditary spastic paraplegia controls ribosome assembly in mitochondria. Cell. 2005;123(2):277–289. 16239145 10.1016/j.cell.2005.08.003

[5] Koppen M, Langer T. Protein degradation within mitochondria: Versatile activities of AAA proteases and other peptidases. Crit Rev Biochem Mol Biol. 2007;42(3):221–242. 17562452 10.1080/10409230701380452

[6] Pareek G, Thomas RE, Pallanck LJ. Loss of the Drosophilam-AAA mitochondrial protease paraplegin results in mitochondrial dysfunction, shortened lifespan, and neuronal and muscular degeneration. Cell Death Dis. 2018;9(3):304. 29467464 10.1038/s41419-018-0365-8 PMC5833341

[7] Ferreirinha F, Quattrini A, Pirozzi M, et al Axonal degeneration in paraplegin-deficient mice is associated with abnormal mitochondria and impairment of axonal transport. J Clin Invest. 2004;113(2):231–242. 14722615 10.1172/JCI20138 PMC311437

[8] Damiani D, Baggiani M, Della Vecchia S, Naef V, Santorelli FM. Pluripotent stem cells as a preclinical cellular model for studying hereditary spastic paraplegias. Int J Mol Sci. 2024;25(5):2615. 38473862 10.3390/ijms25052615 PMC10932093

[9] Shi Y, Kirwan P, Livesey FJ. Directed differentiation of human pluripotent stem cells to cerebral cortex neurons and neural networks. Nat Protoc. 2012;7(10):1836–1846. 22976355 10.1038/nprot.2012.116

[10] Brazdis R-M, Alecu JE, Marsch D, et al Demonstration of brain region-specific neuronal vulnerability in human iPSC-based model of familial Parkinson’s disease. Hum Mol Genet. 2020;29(7):1180–1191. 32160287 10.1093/hmg/ddaa039 PMC7206857

[11] Giorgio V, von Stockum S, Antoniel M, et al Dimers of mitochondrial ATP synthase form the permeability transition pore. Proc Natl Acad Sci U S A. 2013;110(15):5887–5892. 23530243 10.1073/pnas.1217823110 PMC3625323

[12] Sambri I, Massa F, Gullo F, et al Impaired flickering of the permeability transition pore causes SPG7 spastic paraplegia. Ebiomedicine. 2020;61:103050. 33045469 10.1016/j.ebiom.2020.103050 PMC7553352

[13] Wali G, Li Y, Liyanage E, Kumar KR, Day ML, Sue CM. Pharmacological rescue of mitochondrial and neuronal defects in SPG7 hereditary spastic paraplegia patient neurons using high throughput assays. Front Neurosci. 2023;17:1231584. 37766787 10.3389/fnins.2023.1231584 PMC10520970

[14] Wali G, Kumar KR, Liyanage E, Davis RL, Mackay-Sim A, Sue CM. Mitochondrial function in hereditary spastic paraplegia: Deficits in SPG7 but not SPAST patient-derived stem cells. Front Neurosci. 2020;14:820. 32973427 10.3389/fnins.2020.00820 PMC7469654

[15] Atorino L, Silvestri L, Koppen M, et al Loss of m-AAA protease in mitochondria causes complex I deficiency and increased sensitivity to oxidative stress in hereditary spastic paraplegia. J Cell Biol. 2003;163(4):777–787. 14623864 10.1083/jcb.200304112 PMC2173682

[16] Montoro-Gámez C, Nolte H, Molinié T, et al SARM1 deletion delays cerebellar but not spinal cord degeneration in an enhanced mouse model of SPG7 deficiency. Brain J Neurol. 2022;146(10):4117–4131. 10.1093/brain/awad136 37086482

[17] Pfeffer G, Gorman GS, Griffin H, et al Mutations in the SPG7 gene cause chronic progressive external ophthalmoplegia through disordered mitochondrial DNA maintenance. Brain. 2014;137(5):1323–1336. 24727571 10.1093/brain/awu060 PMC3999722

[18] Wedding IM, Koht J, Tran GT, et al Spastic paraplegia type 7 is associated with multiple mitochondrial DNA deletions. PLoS One. 2014;9(1):e86340. 24466038 10.1371/journal.pone.0086340 PMC3899233

[19] Güner F, Pozner T, Krach F, et al Axon-specific mitochondrial pathology in SPG11 alpha motor neurons. Front Neurosci. 2021;15:680572. 34326717 10.3389/fnins.2021.680572 PMC8314181

[20] Havlicek S, Kohl Z, Mishra HK, et al Gene dosage-dependent rescue of HSP neurite defects in SPG4 patients’ neurons. Hum Mol Genet. 2014;23(10):2527–2541. 24381312 10.1093/hmg/ddt644 PMC3990156

[21] Denton K, Mou Y, Xu C-C, et al Impaired mitochondrial dynamics underlie axonal defects in hereditary spastic paraplegias. Hum Mol Genet. 2018;27(14):2517–2530. 29726929 10.1093/hmg/ddy156 PMC6031053

[22] Shanmughapriya S, Rajan S, Hoffman NE, et al SPG7 is an essential and conserved component of the mitochondrial permeability transition pore. Mol Cell. 2015;60(1):47–62. 26387735 10.1016/j.molcel.2015.08.009 PMC4592475

[23] Hurst S, Baggett A, Csordas G, Sheu S-S. SPG7 targets the m-AAA protease complex to process MCU for uniporter assembly, Ca2+ influx, and regulation of mitochondrial permeability transition pore opening. J Biol Chem. 2019;294(28):10807–10818. 31097542 10.1074/jbc.RA118.006443 PMC6635443

[24] Chang KT, Niescier RF, Min K-T. Mitochondrial matrix Ca2+ as an intrinsic signal regulating mitochondrial motility in axons. Proc Natl Acad Sci U S A. 2011;108(37):15456–15461. 21876166 10.1073/pnas.1106862108 PMC3174631

[25] Jung H, Kim SY, Canbakis Cecen FS, Cho Y, Kwon S-K. Dysfunction of mitochondrial Ca2+ regulatory machineries in brain aging and neurodegenerative diseases. Front Cell Dev Biol. 2020;8:599792. 33392190 10.3389/fcell.2020.599792 PMC7775422

[26] Elrod JW, Wong R, Mishra S, et al Cyclophilin D controls mitochondrial pore–dependent Ca2+ exchange, metabolic flexibility, and propensity for heart failure in mice. J Clin Invest. 2010;120(10):3680–3687. 20890047 10.1172/JCI43171 PMC2947235

[27] Bernardi P, Rasola A, Forte M, Lippe G. The mitochondrial permeability transition pore: Channel formation by F-ATP synthase, integration in signal transduction, and role in pathophysiology. Physiol Rev. 2015;95(4):1111–1155. 26269524 10.1152/physrev.00001.2015 PMC4600949

[28] Amanakis G, Murphy E. Cyclophilin D: An integrator of mitochondrial function. Front Physiol. 2020;11:595. 32625108 10.3389/fphys.2020.00595 PMC7311779

[29] Elrod JW, Molkentin JD. Physiologic functions of Cyclophilin D and the mitochondrial permeability transition pore. Circ J. 2013;77(5):1111. 23538482 10.1253/circj.cj-13-0321 PMC6397958

[30] Basso E, Fante L, Fowlkes J, Petronilli V, Forte MA, Bernardi P. Properties of the permeability transition pore in mitochondria devoid of Cyclophilin D*. J Biol Chem. 2005;280(19):18558–18561. 15792954 10.1074/jbc.C500089200

[31] Johnson KM, Chen X, Boitano A, Swenson L, Opipari AW, Glick GD. Identification and validation of the mitochondrial F1F0-ATPase as the molecular target of the immunomodulatory benzodiazepine Bz-423. Chem Biol. 2005;12(4):485–496. 15850986 10.1016/j.chembiol.2005.02.012

[32] Amanakis G, Bustamante M, Sun J, Liu C, Kitsis R, Murphy E. The role of cyclophilin D isomerase activity in regulating the mitochondrial permeability transition pore. J Mol Cell Cardiol. 2022;173:66–67. 10.1016/j.yjmcc.2023.09.003 PMC10809717 37696137

[33] Bhagavathula N, Nerusu KC, Hanosh A, et al 7-Chloro-5-(4-hydroxyphenyl)-1-methyl-3-(naphthalen-2-ylmethyl)-4,5-dihydro-1 H-benzo[b][1,4]diazepin-2(3 H)-one (Bz-423), a benzodiazepine, suppresses keratinocyte proliferation and has antipsoriatic activity in the human skin-severe, combined immunodeficient mouse transplant model. J Pharmacol Exp Ther. 2008;324(3):938–947. 18055879 10.1124/jpet.107.130955

[34] Blatt NB, Boitano AE, Lyssiotis CA, Opipari AW, Glick GD. Bz-423 superoxide signals B cell apoptosis via Mcl-1, Bak, and Bax. Biochem Pharmacol. 2009;78(8):966–973. 19481066 10.1016/j.bcp.2009.05.025 PMC2759512

