# Supplementary material for: Rescue of mitochondrial and neurite pathology in SPG7 hereditary spastic paraplegia patient-derived cortical neurons
Source: Brain Commun. 2026 Jun 25;8(3):fcag219. doi: 10.1093/braincomms/fcag219 (PMC13296996; doi:10.1093/braincomms/fcag219)
Supplement: fcag219_Supplementary_Data [file fcag219_supplementary_data.pdf]

Rescue of mitochondrial and neurite pathology in *SPG7* hereditary spastic paraplegia patient-derived cortical neurons

Neubauer et al.

Supplementary table

Supplemental table 1. Clinical phenotypes and genetic characteristics of *SPG7* patients and controls

Identifier	5vv	h11	jdq	7mn	82a	33q
**Genotype *SPG7***	c.1529C>T, c.958dupG	c.233T>A, c.(183+1_184-1)_(1449+1_1450-1)del	c.1045G>A, c.2115_2131del, frameshift	wildtype	wildtype	wildtype
**Protein change**	p.Ala510Val; frameshift exon 7	p.Leu78X; deletion exons 2–10	p.Gly349Ser; frameshift exon 17			
**Analyzed iPSC lines**	UKERi5vv-002 UKERi5vv-010	UKERih11-001 UKERih11-002	UKERijdq-002 UKERijdq-009	UKERi7mn-010 UKERi7mn-017	UKERi82a-004 UKERi82a-022	UKERi33q-101 UKERi33q-103 UKERi33q-109
**Sex**	female	female	female	female	female	female
**Age at onset /age at examination (years)**	55/63	42/49	40/64	-/52	-/65	-/45
**SPRS (0-52)**	11	21	27	–	–	–
**Lower limb spasticity**	++	++	++	–	–	–
**Maximum gait distance**	2000 meters	50 meters	200 meters	–	–	–
**Pyramidal signs**	+	+	+	–	–	–
**Cerebellar signs**	–	+	+	–	–	–
**Optic atrophy**	–	–	–	–	–	–
**Cognitive impairment**	–	–	–	–	–	–
**Bladder dysfunction**	–	+	+			–
**Additional symptoms**	vertical ocular palsy	–	axonal neuropathy	–	–	–
**MR imaging**	normal	normal	normal	normal	normal	normal

SPRS: spastic paraplegia rating scale; *iPSC*: induced pluripotent stem cells.

Supplementary methods

Generation of iPSC

All participants provided written informed consent, and all experiments received approval from the local institutional review board (approval no. 259_17B: “Biobank zur Untersuchung von Biomarkern und Generierung humaner Zellmodelle bei Neurologischen Erkrankungen”, ethics committee of the Friedrich-Alexander- Universität Erlangen-Nürnberg, Erlangen, Germany). Meeting the minimum standard for exploratory patient vs. control derived iPSC modeling, t his study included three patients with biallelic pathogenic variants in the SPG7 gene and two age- and sex-matched controls (Supplemental Table 1). All study participants were of Caucasian descent, and controls were unrelated to the included patients. The three patients were compound heterozygous for pathogenic variants in the SPG7 gene, and all SPG7 patients carried at least one variant resulting in a premature stop codon or a large deletion. Dermal punch biopsies were conducted to obtain fibroblasts from both patients and control subjects. Subsequently, induced pluripotent stem cells (iPSCs) were generated through non-integrating lentiviral Sendai reprogramming. The Yamanaka transcription factors, specifically the CytoTuneTM-iPS Sendai Reprogramming Kit (Thermo Fisher Scientific), were employed following the manufacturer’s protocol, as previously described.[1] For each individual, a minimum of 20 cell lines were acquired during reprogramming, with two cell lines subsequently expanded for further characterization. Pluripotency of SPG7 and control iPSCs was confirmed by flow cytometry analysis of Tra-1-60 expression (anti-Tra-1-60-R AF488, Biolegend, 330614) (Supplemental Fig. 1A). Detailed information regarding the raw data of specific clones and their utilization in each experiment is provided in the respective supplementary figures.

Copy number variation (CNV) analysis

DNA was extracted from hiPSC lines using the DNeasy Blood and Tissue Kit (Qiagen).
Low-pass whole genome sequencing was performed on a NovaSeq 6000 system, and reads were aligned to an hg19 genomic reference (UCSC hg19 with N-masked ChrY-PARs) using DRAGEN v3.10. The average sequencing depth per sample was approximately 2x.
Copy number analysis was conducted using the DRAGEN CNV pipeline with GC-normalized read counts aggregated in 100 kb bins (instead of the default 1 kb) to account for the low sequencing depth. Because of the high relative variance in read depth, self-normalization was used for CNV calling. CNV segments were subsequently generated from the GC-normalized counts using the DRAGEN v3.10 CNV module.
All downstream computational analyses and data visualization were performed in Python (version 3.10) using custom scripts based on standard scientific libraries. CNV > 1000 kb were analyzed further. Classification into “benign / artefact” was performed by a geneticist when comparing to in-house CNV datasets derived from blood sample genomic DNA.

Cortical neuron differentiation

Control and patient iPSC were differentiated into neural progenitor cells (NPCs) and cortical neurons following an adapted dual SMAD-inhibition based small molecule monolayer protocol .[2] In brief, iPSCs were differentiated in a monolayer in medium containing Neurobasal, DMEM-F12, N-2, B-27 supplements (Thermo Fisher Scientific) and ascorbic acid (50 µM). For 12 days, dual SMAD inhibition was performed with LDN193189 (0.1 µM) and SB431542 (10 µM). The medium was changed daily during the initial 12 days, and afterwards, it was changed every other day. On day 12, cells were dissociated and subsequently expanded in FGF2 (20 ng/ml). Subsequently, cultures were switched to neuronal differentiation medium containing BDNF (20 ng/ml), GDNF (20 ng/ml), cAMP (0.5 ng/ml) and ascorbic acid (200 µM). Analyses were performed 14 days and 28 days after maturation in neuronal differentiation medium. In order

to prevent cell detachment of the high density cultures, culture plates were coated with Geltrex during induction and expansion, and with polyornithine and laminine during differentiation.

Flow cytometry (FC)

For flow cytometry analysis of intracellular markers, NPCs were detached with Accutase for 10 min and washed with FC buffer (2% FCS, 0.01% NaN3, 2mM EDTA in PBS). $5x10^5$ cells were resuspended in 100 µL BD Cytofix/Cytoperm™ Fixation and Permeabilization Solution (BD Biosciences, 554722) and incubated for 10 min at room temperature. Then, 100 µL of Nuclear Permeabilization Solution [NPS, 0.05% Triton X-100 in 1X BD Perm/Wash™ buffer (10X diluted with dH2O) (BD Biosciences, 554723)] were directly added and incubated for additional 5 mins. Cells were washed with NPS and antibodies were incubated for 20 min at room temperature. Antibodies used: anti-Nestin PerCP-Cy5.5 (BD Biosciences, 561231), anti-PAX-6 APC (Miltenyi Biotec, 130-123-267), Anti-Sox2 PE (BD Biosciences, 560291). Afterwards, cells were washed twice with NPS and were measured on a CytoFLEX S flow cytometer (Beckman Coulter). Data analysis was done with the accompanying CytExpert software (version 2.4.0.28).

Immunofluorescence (IF)

Cortical neurons were cultured on glass cover slips, coated with polyornithine and laminine, in 24 well plates. After 14 days or 28 days of differentiation, respectively, two thirds of medium was replaced by 4% paraformaldehyde in PBS and incubated for 15 minutes at room temperature. Subsequently, neurons were washed thrice with PBS. Neurons were permeabilized and blocked with 0.1% Triton X-100 diluted in 3% donkey serum for 30 minutes at room temperature. Respective primary antibodies were incubated overnight at 4 °C or for 3 h at room temperature, diluted in 3% donkey serum in PBS. Afterwards, cells were washed thrice with PBS and incubated with the secondary antibodies (Alexa Fluor® probes) for 1 hour at room temperature in the dark. As nuclear counterstain, DAPI was incubated at a concentration of 1:10,000 in 3% donkey serum in PBS for 3 minutes at room temperature. Cells were again washed thrice with PBS, before mounting them on glass slides with Aqua Polymount and drying overnight at room temperature. Imaging was performed on an Axio Observer.Z1 (Carl Zeiss) with Apotome using both a 20x objective and a 63x objective with oil Immersion. The following antibodies were used for IF: beta3-tubulin (Tuj1; rabbit polyclonal, 1:250, Abcam, ab18207), ctip2 (rat, 1:500, Abcam, ab18465).

Western Blots

Protein expression was quantified by Western blot. Cells were cultured in 6-well plates, at a seeding density of 2 million NPCs per well. Medium changes were performed every other day. Before cells were lysed, they were washed three times with PBS to remove remaining medium. Protein was extracted in RIPA buffer [50mM Tris-HCl, pH 8.0, 150mM NaCl, 1% v/v Nonidet P-40 (NP-40; Roche, 11754599001), 0.5% v/v Na-deoxycholate (Sigma-Aldrich, D6750), 0.1% w/v SDS (Applichem, 765 A1112,0100), 2mM EDTA, with protease (Roche, 11836170001) and phosphatase (Roche, 04906837001) inhibitors]. Protein concentration was measured with a BCA assay and lysates were diluted to the desired protein concentration. Laemmli buffer was added before cells were heated at 95°C for 5 minutes. Approximately 30 µg of total protein was separated on 4–12% Bis-Tris gels (Invitrogen, Life Technologies) and blotted onto PVDF membrane (Immobilon-P, Millipore, IPVFL00010). Membranes were incubated at room temperature (RT) for 1 h in PBS with 0.1% Tween-20 (Applichem, A1389,0500) and 1% w/v bovine serum albumin (BSA; Sigma-Aldrich, A9647) as a blocking agent (PBST-BSA), followed by primary antibody incubation overnight at 4 °C with mild shaking. After washing with PBST, membranes were probed with appropriate secondary antibodies. Bands were visualized with ChemiDoc Imaging System (Bio-Rad). Protein expression was quantified by

normalization to GAPDH expression using ImageJ. The following antibodies were used: paraplegin (mouse monoclonal, 1:500, Santa Cruz, sc-514393), GAPDH (goat, 1:2500, R&D, AF5718).

Analysis of axonal outgrowth

To individually label cortical neurons, we employed the transfection method with low amounts of the plasmid pEF1-dTomato. Neurons underwent transfection either on day 5 or day 12 of differentiation using Lipofectamine (LTX, ThermoFisher) and were fixed 48 hours later. Subsequently, neurons were counterstained using immunofluorescence (IF) for the expression of beta3-tubulin, CTIP2, and DAPI, and were then mounted on slides. Complete neurite outlines of dTomato/CTIP2 double-positive cells only were captured using the Observer.Z1 fluorescence microscope (Zeiss) and included in the analysis. Neurites were traced using the ImageJ Plugin NeuronJ and served as the basis for Sholl analysis. Total neurite length represents the sum of all neurite lengths of a single CTIP2-positive neuron. Primary neurites originate from the cell soma, while secondary neurites extend from primary neurites. Sholl analysis utilized the 'Sholl analysis' Plugin for ImageJ. The center of the soma was manually marked as the Sholl starting point, with the longest neurite as the endpoint. Circles of increasing radius in 10 μm increments were analyzed from the starting point, quantifying the number of neurite intersections at each radius.

Microfluidic chambers

To record and analyze directional axonal transport and morphology of mitochondria along neurites, neurons were cultivated in microfluidic chambers as described previously.[3] In short, microfluidic silicone chambers consist of two compartments (soma and axonal side), separated by axonal grooves of 450 μm length (SND450, Xona Microfluidics). 80,000 NPCs alongside 10,000 human cerebellar astrocytes (HA-c, ScienCell) were seeded into the soma side and 20,000 astrocytes were seeded into the axonal side. After seeding, differentiation into cortical neurons was performed using the above mentioned differentiation protocol. At a multiplicity of infection of 1, n eurons were infected with a mitoDsRed lentiviral construct two days post seeding to label mitochondria. After 2 days of lentivirus incubation, medium was changed and microfluidic chambers were washed once with N2/B27 medium. After 14 and 28 days of differentiation, respectively, neuritic growth into the microgrooves was assessed by microscopy. Grooves were recorded for a total of 10 minutes, taking an immunofluorescence image every 5 seconds. By manually tracing mitochondrial movement throughout the recording, kymographs of neuritic mitochondria transport with a horizontally displayed neurite length of 200 μm and vertically displayed recording duration of 600 seconds were created. In total, two microfluidic chambers were set up and at least 8 kymographs were created per sample. Mitochondrial transport velocities were analyzed by manually measuring mitochondria displacement in the respective kymograph. Track displacement was set to at least 3 μm and mitochondria visibility had to exceed one-third of recording duration. Mitochondrial movements were subdivided into antero- and retrograde. Transport velocities were then calculated as the average velocity throughout the movement.

Electron microscopy

Ultrastructural images of mitochondria were acquired by transmission electron microscopy. In brief, 500,000 NPC were seeded onto 24 well plates coated with Geltrex. Medium change was performed every other day. After 14 days of differentiation, medium was aspirated, cells were washed one time with PBS and tissue was fixed in Itho-buffer, which contains 3% glutaraldehyde (GA). Embedding in raisin and sectioning was performed as described before.[4] All ultrathin sections were transferred into a JEOL 1400Plus transmission electron microscope operating at 120kV.

Mitochondrial morphology in distal neurites

Neuritic mitochondrial morphology in the very distal compartment of mature cortical neurons was assessed within the axonal side of microfluidic chamber cultures (see above). Medium was changed once per week and imaging was performed after 50 days of neuronal differentiation. Images of distal neurites were taken at the Zeiss Axio Observer.Z1 at a 63x magnification. Morphology was analyzed by manual outlining of 80-100 mitochondria per cell line within at least two microfluidic chambers per cell line, in ImageJ. Perimeter, area, aspect ratio (i.e., ratio of maximum and minimum diameter of mitochondria), and circularity were determined.

Apoptosis assay

To quantify apoptosis induction by Bz-423 treatment, the eBioscience Annexin V FITC Apoptosis detection kit was used according to manufacturer's instructions (Thermo Fisher, BMS500FI-100). Briefly, on day 7 of differentiation, dead cells in supernatants were collected and cortical neurons were detached with Accutase. 5x10^5/mL cells were resuspended in 1X binding buffer, and Annexin V FITC (1:40) was added for 10 mins at room temperature. The cells were washed with 1X binding buffer, and propidium iodide (1:20) was added directly before measurement on a MACSQuant Analyzer 16 (Miltenyi Biotec). For data analysis, the MACSQuantify software (version 3.0.2) was used. Gates were set according to the fluorescence minus one (FMO) controls, respectively.

Supplementary figures

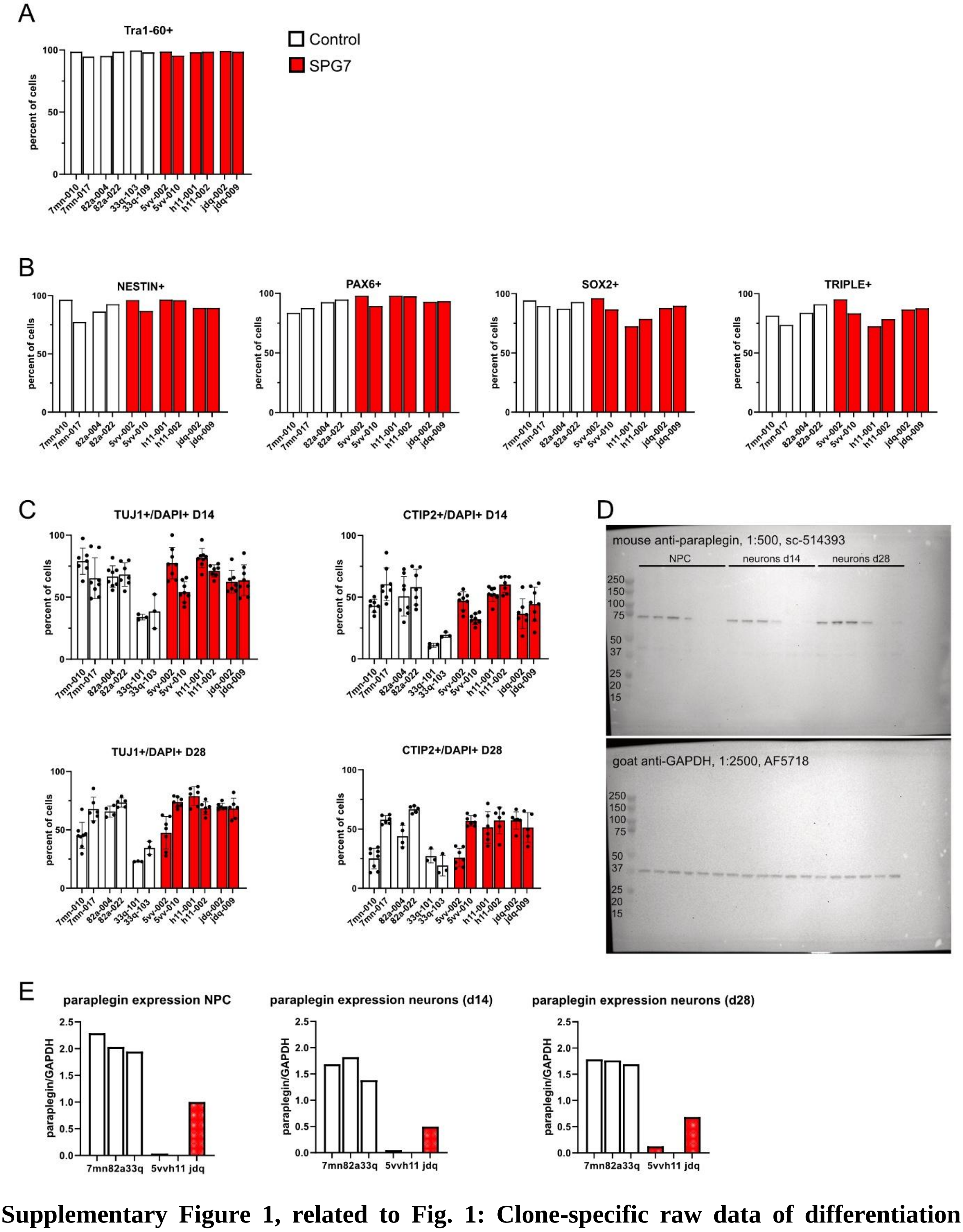

Supplementary Figure 1, related to Fig. 1: Clone-specific raw data of differentiation analyses. (A) Analysis of iPSC clones for Tra-1-60 expression. (B) Flow cytometry analysis of NESTIN, PAX6, SOX2 positive cells, and NESTIN/SOX2/PAX6 triple-positive cells. (C) Immunocytochemistry analyses of cells positive for TUJ1 and CTIP2 relative to the total number of DAPI-positive cells. Each dot represents the ratio per randomly chosen field of view. Per clone, at least 6 fields of view were analyzed, obtained from at least two experimental replicates. (D) Full Western Blots for Paraplegin and GAPDH shown in Fig. 1E. (E) Western

Blot analysis of NPC and neurons (after 14 and 28 days of neuronal differentiation) for expression of Paraplegin, obtained from one experiment.

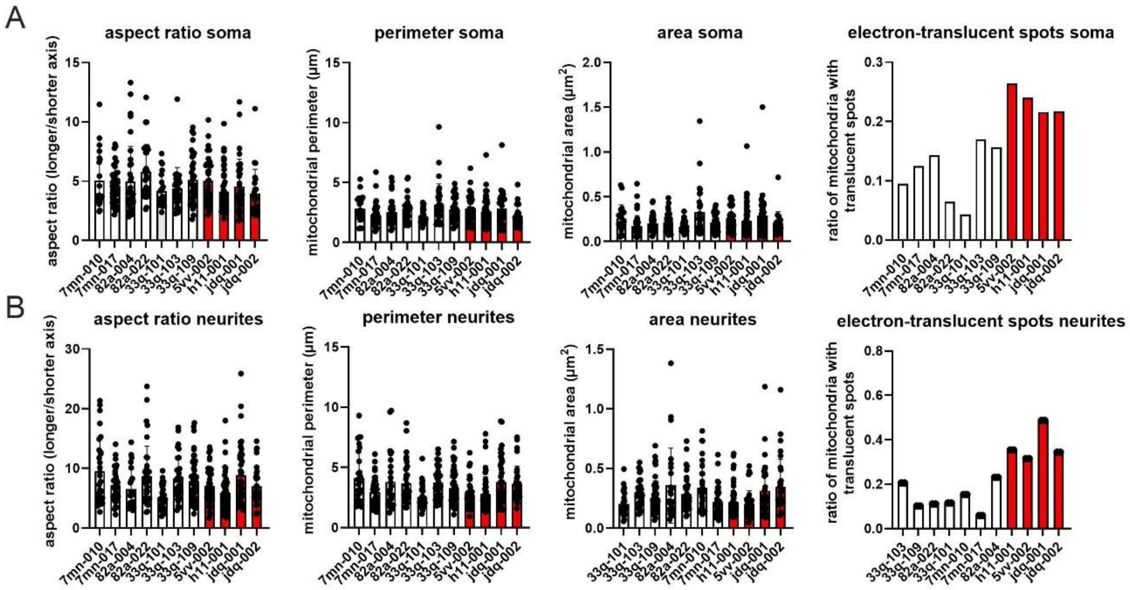

Supplementary Figure 2, refers to Fig. 2: Clone-specific raw data of mitochondrial analyses. (A-B) Clone-specific measures of ultrastructural morphology of mitochondria located in the soma (A) and in neurites (B). Each dot represents parameters obtained from one mitochondrion. Ratios of mitochondria with translucent spots were obtained at two experimental timepoints.

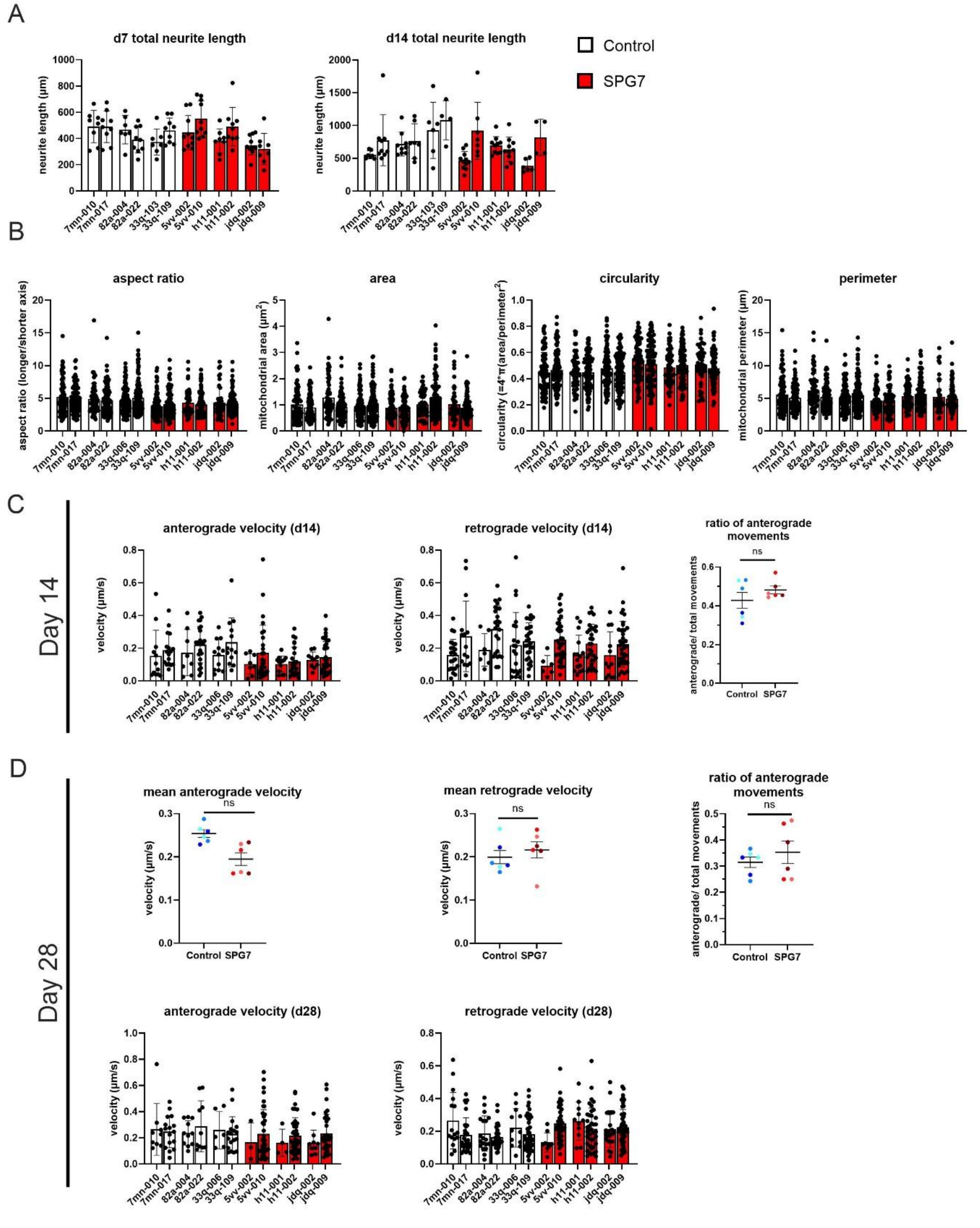

Supplementary Fig. 3, refers to Fig. 3. Clone-specific raw data of neurite length and axonal transport analyses. (A) Raw data per iPSC clone, each dot represents neurite length of one cortical neuron, obtained from at least two replicates. (B) Distal mitochondrial morphology raw data per iPSC clone. Each dot represents one mitochondrion. Per clone, at least two replicate microfluidic chambers were analyzed, obtained at two experimental timepoints. (C) Mitochondrial axonal transport raw data per iPSC clone (left and middle panel) at 14 days of differentiation. Each dot represents one mitochondrion. Per clone, at least two replicate microfluidic chamber setups were analyzed, obtained at the same timepoints as the distal mitochondrial morphology analysis. Right panel: ratio of anterogradely moving mitochondria

over all moving mitochondria. Groups were compared by a linear mixed-effects model. $n_{Control}$=6 iPSC clones derived from 3 donors, n_{SPG7}=6 iPSC clones derived from 3 donors. Per clone, at least 10 mitochondria from 2 microfluidic chambers were quantified. Each dot represents the mean of one iPSC clone, each donor is coded by a separate color. *ns* not significant. (D) Quantification of retrograde mitochondrial transport velocity at 28 days of differentiation. Groups were compared by linear mixed-effects models. $n_{Control}$=6 iPSC clones derived from 3 donors, n_{SPG7}=6 iPSC clones derived from 3 donors. Per clone, at least 10 mitochondria from 2 microfluidic chambers were quantified, obtained at two experimental timepoints. Upper line: Each dot represents the mean of one iPSC clone, each donor is coded by a separate color. Lower line: raw data per iPSC clone. *ns* not significant; * $P<0.05$; *** $P<0.001$.

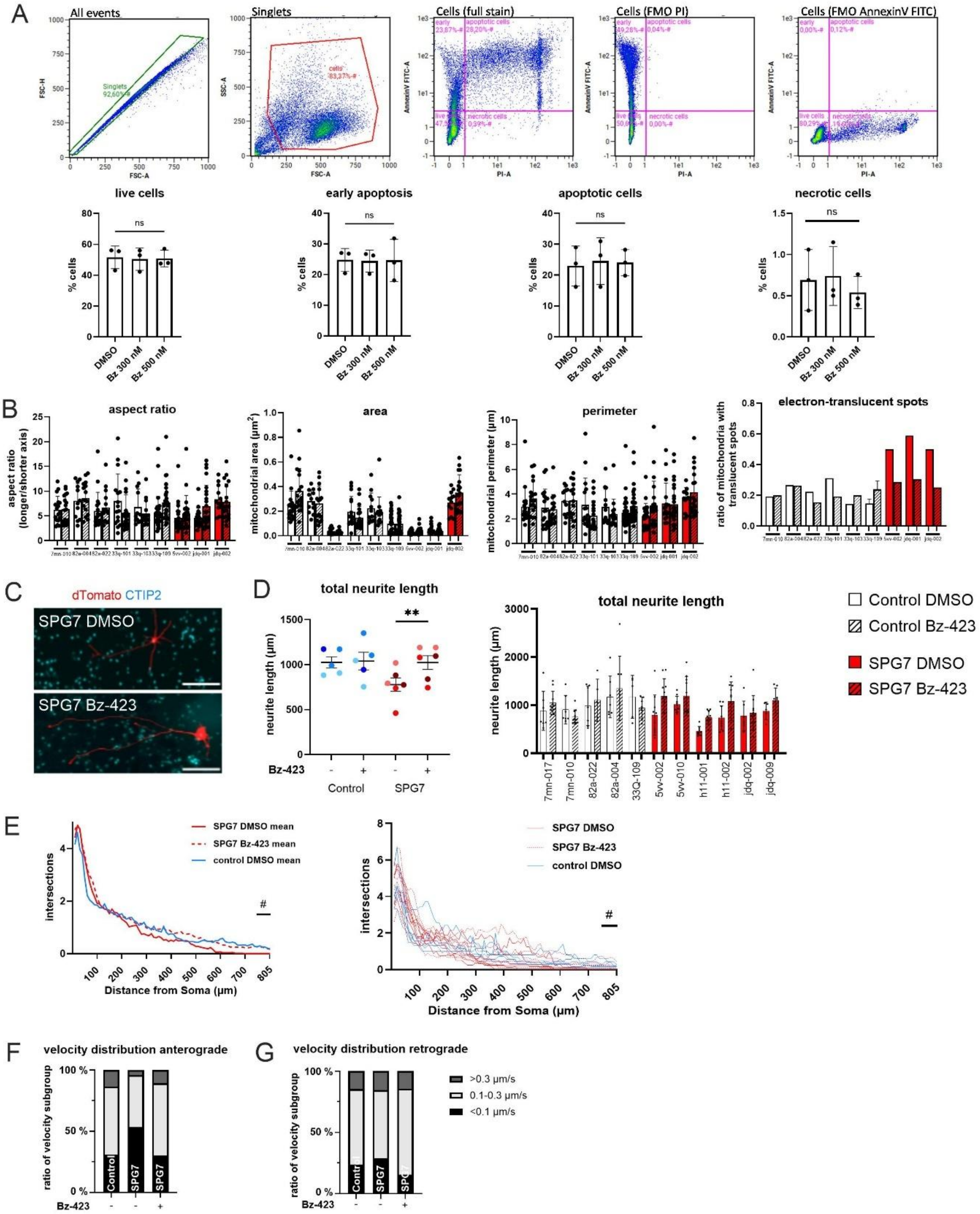

Supplementary Fig. 4, refers to Fig. 4. Clone-specific raw data of pharmacological treatment experiments. (A) Gating strategy of flow cytometric apoptosis assay demonstrated on the raw measurement data of one control line (upper line). Each dot represents a single cell detection event. Cortical neurons from three control lines were treated every other day with vehicle (DMSO), 300 nM Bz-423 or 500 nM Bz-423 during differentiation over the course of 7 days. Gating was set according to respective FMOs. Quantifications (lower line) of live cells (Annexin V negative, PI negative), early apoptotic cells (Annexin V positive, PI negative), apoptotic cells (Annexin V positive, PI positive) and necrotic cells (Annexin V negative, PI positive) are shown as percentage of total cells. Each dot represents the mean of at least 1,000 cells that were analyzed by flow cytometry per line. Groups were compared by a Kruskal-Wallis test. *ns* not significant. (B) Raw quantification data of ultrastructural mitochondrial morphology

analyses. Each dot represents one mitochondrion. At least two replicates were analyzed per condition. (C) Representative images of dTomato expressing and CTIP2 positive SPG7 cortical neurons, treated with DMSO (upper image) or Bz-423 (lower image). Scale bars 100 µm. (D) Quantification of total neurite length (left panel) and underlying raw counts (right panel). SPG7 DMSO vs. SPG7 Bz-423 was compared using a linear mixed-effects models with Tukey adjustment for between-group comparisons. $n_{Control}$=5 iPSC clones derived from 3 donors (per treatment condition), n_{SPG7}=6 iPSC clones derived from 3 donors (per treatment condition). Per clone and treatment condition, complete neurite morphology from at least 8 neurons was quantified. Each dot represents results from one iPSC clone. Each donor is coded by a separate color. * $P<0.05$. (E) Sholl analysis of SPG7-DMSO vs. SPG7 Bz-423, analyzed on the same neurite tracing image dataset as in (D). Left diagram shows means for each condition; right diagram shows means for each iPSC clone. Mixed-effects model analyses were performed comparing SPG7 DMSO and SPG7 Bz-423 at each distance. $^{\#}$ $P<0.05$. (F) Distribution of anterograde transport velocities. (G) Distribution of retrograde transport velocities. *FMO* fluorescence minus one.

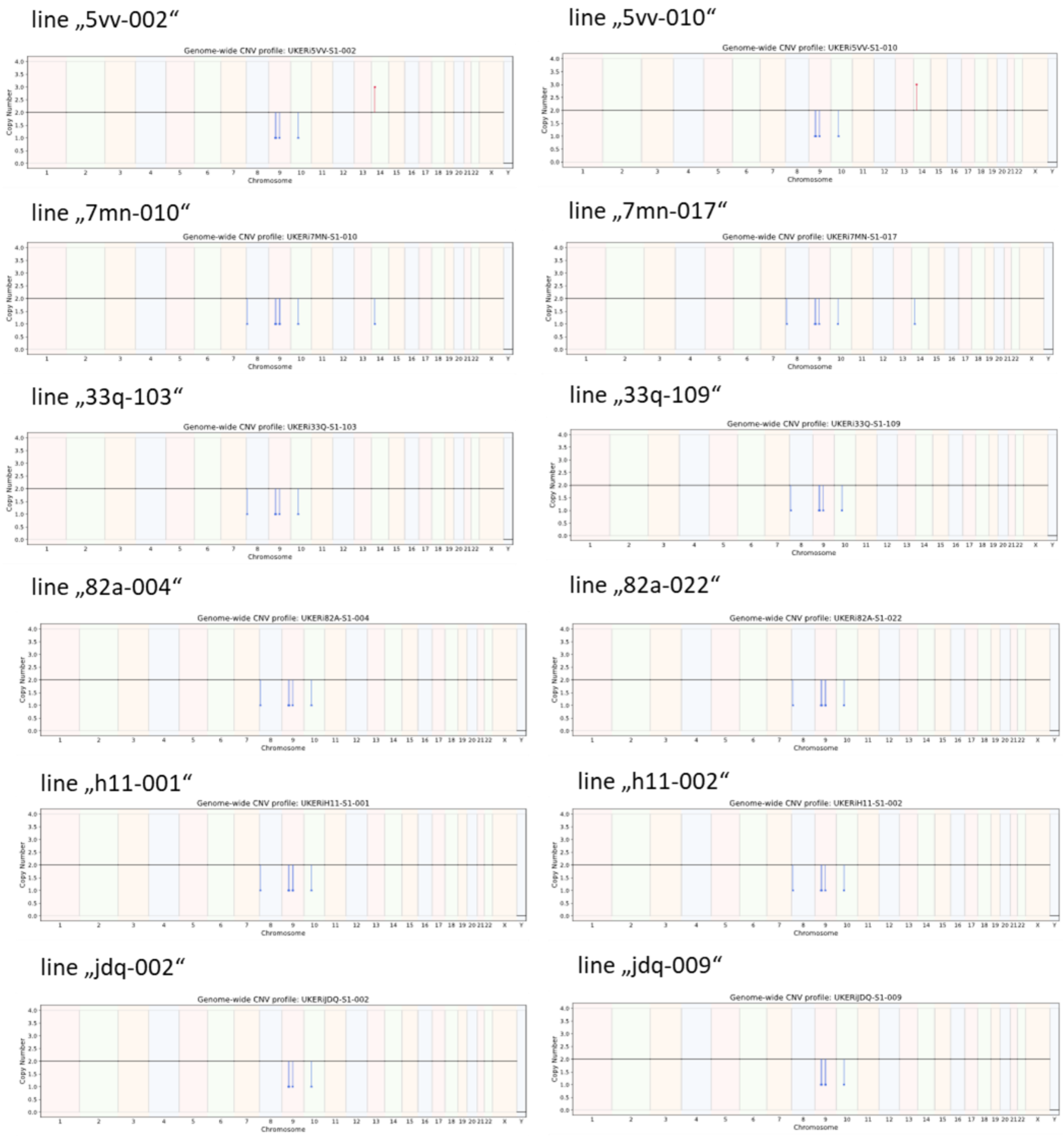

Supplementary Fig. 5. Copy number variation analysis. Landscape plots of the copy number variation analysis of iPSC lines that were utilized in this study. When comparing to control data, all detected losses and gains were classified as “benign / artefact”.

Supplementary references

1. Popp B, Krumbiegel M, Grosch J, et al. Need for high-resolution Genetic Analysis in iPSC: Results and Lessons from the ForIPS Consortium. Sci Rep-uk. 2018;8(1):17201. doi:10.1038/s41598-018-35506-0
2. Turan S, Boerstler T, Kavyanifar A, et al. A novel human stem cell model for Coffin–Siris syndrome-like syndrome reveals the importance of SOX11 dosage for neuronal differentiation and survival. Hum Mol Genet. 2019;28(15):2589-2599. doi:10.1093/hmg/ddz089
3. Güner F, Pozner T, Krach F, et al. Axon-Specific Mitochondrial Pathology in SPG11 Alpha Motor Neurons. Front Neurosci. 2021;15:680572. doi:10.3389/fnins.2021.680572
4. Asano N, Hampel U, Garreis F, et al. Differentiation Patterns of Immortalized Human Meibomian Gland Epithelial Cells in Three-Dimensional Culture. Investig Opthalmology Vis Sci. 2018;59(3):1343. doi:10.1167/iovs.17-23266
